# Carbon upshift in *Lactococcus cremoris* elicits immediate initiation of proteome-wide adaptation, coinciding with growth acceleration and pyruvate dissipation switching

**DOI:** 10.1128/mbio.02990-24

**Published:** 2025-02-20

**Authors:** Berdien van Olst, Sjef Boeren, Jacques Vervoort, Michiel Kleerebezem

**Affiliations:** 1 Host-Microbe Interactomics, Wageningen University & Research, Wageningen, The Netherlands; 2 Laboratory of Biochemistry, Wageningen University & Research, Wageningen, The Netherlands; 3 TI Food and Nutrition, Wageningen, The Netherlands; The University of Queensland, Brisbane, Queensland, Australia

**Keywords:** *Lactococcus*, proteomics, environmental adaptation, carbon metabolism

## Abstract

Fitness optimization in a dynamic environment requires bacteria to adapt their proteome in a tightly regulated manner by altering protein production and/or degradation. Here, we investigate proteome adaptation in *Lactococcus cremoris* following a sudden nutrient upshift (e.g., nutrients that allow faster growth) and focus especially on the fate of redundant proteins after the shift. Protein turnover analysis demonstrated that *L. cremoris* cultures shifted from galactose to glucose, immediately accelerate growth and initiate proteome-wide adjustment toward glucose-optimized composition. Redundant proteins were predominantly adjusted by lowering (or stopping) protein production combined with dilution by growth. However, pyruvate formate lyase activator (PflA) was actively degraded, which appears correlated to reduced 4Fe-4S cofactor availability. Active PflA removal induces the shutdown of galactose-associated mixed acid fermentation to accelerate the switch toward glucose-associated homolactic fermentation. Our work deciphers molecular adjustments upon environmental change that drive physiological adaptation, including growth rate and central energy metabolism.

**IMPORTANCE:**

Bacteria adapt to their environment by adjusting their molecular makeup, in particular their proteome, which ensures fitness optimization under the newly encountered environmental condition. We present a detailed analysis of proteome adaptation kinetics in *Lactococcus cremoris* following its acute transition from galactose to glucose media, as an example of a sudden nutrient quality upshift. Analysis of the replacement times of individual proteins after the nutrient upshift established that the entire proteome is instantly adjusting to the new condition, which coincides with immediate growth rate acceleration and metabolic adaptation. The latter is driven by the active removal of the pyruvate formate lyase activator protein that is pivotal in controlling pyruvate dissipation in *L. cremoris*. Our work exemplifies the amazing rate of molecular adaptation in bacteria that underlies physiological adjustments, including growth rate and carbon metabolism. This mechanistic study contributes to our understanding of adaptation in *L. cremoris* during the dynamic conditions it encounters during (industrial) fermentation, even though environmental transitions in these processes are mostly more gradual than the acute shift studied here.

## INTRODUCTION

Bacteria often face nutritional changes or other physical-chemical shifts in their environment that can affect their growth. Following such environmental shifts, bacteria adapt their proteome to optimize their growth and fitness in the newly encountered environment. Proteome composition and its adaptation are constrained by limitations in the protein synthesis capacity (1, 2). These limitations arise from the limited availability of various cellular resources, ranging from energy and amino acids to ribosome capacity. Moreover, these resources as well as the synthesized proteins occupy cellular space that is also limited (2–5). Consequently, the production of new proteins during proteome adjustment is proposed to come at the cost of other proteins. This requires protein expression to be strictly controlled under dynamic conditions to ensure the optimized production of the right proteins at the appropriate concentration (6) and to prevent the expression of redundant proteins and/or non-optimal protein production levels that have been implied to undermine fitness (2, 6). Therefore, during an environmental shift, proteins that are redundant or non-optimally expressed for the new environment are expected to disappear or be expressed at an adjusted level. Protein levels result from a delicate balance between protein production and protein removal. Protein production is predominantly determined by mRNA abundances, which results from gene transcription and mRNA decay (7). Gene transcription is strictly controlled by a variety of regulatory mechanisms and strongly controls protein production adaptation following an environmental shift. A regulatory mechanism that depends on the interaction of a regulatory protein and an inducer molecule can elicit an immediate protein production arrest when the inducer molecule gets below the threshold level. Such an immediate production stop has been proposed to be the optimal strategy for fast adaptation after an environmental transition (8). However, a gradual downregulation of redundant protein production has been demonstrated in *Escherichia coli* where the lactose-metabolizing proteins continued to be produced after lactose was removed, attributable to the continued availability of the intracellular inducer molecule that controls the transcription regulator LacR (allolactose [9]) and the long lifespan of the lac-operon transcript after the lactose removal (10).

Besides altering protein production, the proteome can also be adjusted by altering protein removal. In steady-state growth conditions, proteins mainly dilute through growth and are not actively degraded (3, 11–15). Moreover, redundant proteins in *Escherichia coli* mainly dilute by growth after the addition of a sugar that supports faster growth (nutrient upshift) or when the sugar supporting faster growth is depleted (nutrient downshift), as well as in nutrient-fluctuating conditions (10, 16). Nevertheless, cells can actively degrade proteins, which is mainly mediated by the Clp and Lon proteases and require energy and accurate recognition of proteins that are targeted for active degradation (17). Active degradation appears to be mainly employed on incorrectly translated proteins or proteins that unfolded due to stress, co-factor loss, or endoproteolytic cleavage (18, 19). Taken together, the cell can adapt its proteome composition after an environmental shift in various ways that include adjusting the *de novo* synthesis of proteins, as well as passive (dilution by growth) or active removal of redundant proteins.

Proteome-wide adaptation after an environmental shift is often inferred by proteome comparisons of cultures that are adapted to distinct environments, which adequately reveals which protein levels change but does not unravel the rate or mechanism by which cells orchestrate these changes during an environmental transition. Studies that investigated these adaptation strategies during a nutrient-quality shift predominantly focused on a small set of specific proteins (10). Moreover, these studies often had the old nutrient still available in the medium after a nutrient-quality upshift or relied on the depletion of the higher-quality nutrient in case of a nutrient-quality downshift, which allows for a transition phase (16). Thereby, these studies might have missed proposed sequential alterations of the proteome (8), missed alternative adaptation strategies for adjusting specific protein levels, and/or might have been confounded by interference effects of other available nutrients.

Here, we investigate how *Lactococcus cremoris* accommodates protein-level changes during the adjustment from growth on galactose to glucose, focusing particularly on the fate of proteins that become redundant. Our initial analyses confirm that the well-established physiological differences of galactose- and glucose-adapted cultures are reflected by their differential proteome composition. Subsequently, we investigate how the proteome is adjusted following an acute shift from galactose to glucose growth by determining protein turnover during a nutrient upshift from galactose to glucose media. Our results establish that growth instantly accelerates upon the shift to glucose, coinciding with an immediate initiation of proteome-wide adjustment. Most redundant proteins are adjusted by changed protein production rates combined with dilution by growth, although specific pyruvate dissipation-associated proteins were actively degraded. In particular, the pyruvate formate lyase activator function (PflA) is rapidly degraded, indicating that *L. cremoris* actively shuts down the galactose-growth-associated mixed acid production to revert to homolactic fermentation that is typically seen during growth on glucose. These findings illustrate that *L. cremoris* employs different strategies to adjust specific categories of redundant protein levels and provides further insight into the critical role of pyruvate dissipation in the central energy metabolism pathway in this organism.

## MATERIALS AND METHODS

### Strains and cultivation

*Lactococcus cremoris* NCDO712 and its plasmid-free derivative MG1363 (20) were grown in batch cultures at 30°C without aeration, using the previously described chemically defined medium for prolonged cultivation (CDMpc) (21, 22). CDMpc was supplemented with 25 mM glucose or 25 mM galactose, and cultures were adapted to these conditions for more than 20 generations by repeated subculturing.

### Growth rate measurements

The growth rate of the adapted cultures was determined by optical density measurements at 600 nm (OD_600_) taken every 30 minutes. Maximal growth rates were calculated from the slope of the linear part of the log-transformed optical density over time.

### Harvesting cells for long-term adaptation

The adapted cultures were diluted 100-fold from early-stationary cultures (OD_600_ ~ 1) into fresh medium and grown to the early logarithmic phase (OD_600_ between 0.3 and 0.4 for glucose conditions and NCDO712 galactose; OD_600_ 0.15 for MG1363 galactose). Samples were collected in biological duplicate (NCDO712; 14 mL) or triplicate (MG1363; 35 mL), and cells were harvested by centrifuging the culture in a swingout centrifuge at 6,520 × *g* for 10 minutes at 4°C. The culture supernatant was transferred to a new tube, and both supernatant and bacterial pellet were flash frozen in N_2_ (l) and stored at −80°C until further use.

### Harvesting cells during short-term environmental adaptation

NCDO712 and MG1363 adapted on CDMpc containing ^12^C^14^N (light) lysine supplemented with 25 mM galactose were inoculated in 100 mL Scott bottles from an overnight culture and grown without aeration at 30°C for at least 10 generations until an OD of 0.2 was reached. Time point zero (t0) was harvested in duplicate by transferring 5 mL culture to a 5 mL Eppendorf and centrifuging at 18,514 × *g* for 3 minutes at 4°C. The culture supernatant was discarded, and the bacterial pellet was flash frozen in N_2_(l) and stored at −80°C until further use. The remaining culture was split into two equal portions, and cells were harvested by centrifuging using a swingout rotor at 3,220 × *g* for 10 minutes at room temperature. The two cell pellets obtained were resuspended in 50 mL of preheated (30°C) CDMpc containing ^13^C^15^N (heavy) lysine supplemented with either 25 mM glucose (“carbon shift”) or 25 mM galactose (“control shift”), respectively. Growth of both cultures was continued at 30°C without aeration, and cells were harvested from 5 mL samples collected every 15 minutes (glucose cultures) or every 30 minutes (galactose cultures) by centrifugation (18,514 × *g*, 3 minutes, 4°C) followed by flash freezing the bacterial pellets in N_2_(l). Samples were stored at −80°C until further use. Sample collection was continued during the first 2 hours (glucose cultures) or first 4 hours (galactose cultures) following the shift to the media containing the heavy lysine, allowing the cultures to grow at least one generation on the post-shift medium and the sampling of eight replicate measurements from the same culture over time.

### Extracellular metabolite measurements

The supernatant of the long-term adapted cultures was used to determine the abundance of extracellular metabolites by nuclear magnetic resonance (NMR) spectroscopy. Samples were diluted 1:1 in a 10% D_2_O/90% H_2_O solution before measuring 1D nuclear Overhauser effect spectroscopy spectra on a 600 MHz NMR (Bruker BioSpin) equipped with a 5 mm cryo-probe at a temperature of 300 K. During relaxation delay and mixing (1H-1H mixing time was 10 ms), 50 Hz saturation on water was applied. The obtained spectra were analyzed with the quantum mechanics-based 1H full spin analysis in the Cosmic Truth software (NMR Solutions Limited) to identify and quantify the peaks (23, 24). The relative peak intensities of acetate, lactate, and ornithine were normalized with the total peak intensity of all peaks. To identify the fermentation pattern, the ratio of acetate and lactate was calculated and rescaled between zero and one, whereas for the ornithine production, the relative peak intensity was normalized against the cell density as determined by OD_600_.

### Proteome analysis

Bacterial pellets were resuspended in ice-cold 100 mM Tris, pH 8, at a concentration of approximately 8 × 10^9^ cells/mL. Cells were lysed (100 µL) using a needle sonicator (MSE) for three times 10 seconds at 22 microns amplitude, with 5 seconds of intermittent cooling on ice. Of the cell lysate, 60 µL was reduced by the addition of 15 mM dithiothreitol and incubation for 30 minutes at 45°C in an Eppendorf Thermomixer Comfort (350 rpm). The remaining lysate was centrifuged to remove cell debris as that could interfere with the BCA assay and was used for a rough protein concentration determination using the BCA assay (Thermo Pierce), according to the manufacturer’s instructions. The reduced lysate sample was denatured and alkylated with 132 µL (long-term adapted cells) or 102 µL (short-term adapted cells) of 8 M urea in 100 mM Tris, pH 8, and a final concentration of 20 mM acrylamide for 10 minutes at 21°C. The alkylated sample was transferred to an ethanol-washed Pall 3K Omega filter (Sigma-Aldrich) and centrifuged for 35 minutes at 13,523 × *g* at room temperature. The proteins on the filter were washed with 130 µL of 50 mM ammonium bicarbonate and centrifuged again for 35 minutes. The washed protein sample was digested overnight on a filter by adding 100 µL of 5 ng/µL sequencing-grade trypsin (Roche) at room temperature. The digested peptides were eluted through the filter membrane by centrifugation for 30 minutes, and the filter was washed with 100 µL of 0.1% HCOOH in water and eluted by centrifugation for 30 minutes. The pH of the final elute was adjusted to pH 3 using 10% trifluoroacetic acid, and this peptide sample was stored at −20°C until further use.

Three or five microliters of peptide samples (based on the peptide intensity measured by a test run) was loaded directly onto a 0.10 × 250 mm ReproSil-Pur 120 C18-AQ 1.9 µm beads analytical column (prepared in-house) at a constant pressure of 825 bar (flow rate of circa 600 nL/minute) with 0.1% HCOOH in water and eluted at a flow of 0.5 µL/minute with a 50 minute linear gradient from 9% to 34% acetonitrile in 0.1% HCOOH using a Thermo EASY nanoLC1000. An electrospray potential of 3.5 kV was applied directly to the eluent via a stainless-steel needle fitted into the waste line of a micro-cross that was connected between the nanoLC and the analytical column. A NanoBlow (25) was installed to prevent ions from entering the MS during equilibration, loading the sample, and for the first 5 minutes after injection. Full scan positive mode FTMS spectra were measured between *m*/*z* 380 and 1,400 on a Q-Exactive HF-X (Thermo electron, San Jose, CA, USA) in the Orbitrap at resolution (60,000). MS and MSMS AGC targets were set to 3 × 10^6^ and 50,000, respectively, or maximum ion injection times of 50 ms (MS) and 25 ms (MSMS) were used. HCD fragmented (isolation width 1.2 *m*/*z*, 24% normalized collision energy) MSMS scans of the 25 most abundant 2–5+ charged peaks in the MS scan were recorded in data-dependent mode (resolution 15,000, threshold 1.2e5, 15 second exclusion duration for the selected *m*/*z* ± 10 ppm).

The obtained MSMS spectra were analyzed with MaxQuant (version 1.6.3.4) (26). Raw data files were searched against the *Lactococcus cremoris* MG1363 database supplemented with NCDO712 plasmid proteins as well as a contaminants database with frequently observed contaminants, such as trypsins (P00760, bovine and P00761, porcine) and human keratins (keratin K22E [P35908], keratin K1C9 [P35527], keratin K2C1 [P04264], and keratin K1CI [P35527]). The digestion mode was set to trypsin/P with a maximum of two missed cleavages, the fixed cysteine modification was set to propionamide, and variable modifications were set to methionine oxidation, protein N-terminal acetylation, and asparagine or glutamine deamidation. A 1% false discovery rate at both peptide and protein levels was allowed; the minimum protein length was set at seven amino acids, and other settings were set as default (27).

For short-term adaptation proteomics, two MaxQuant search runs were performed to quantify proteins only based on their lysine-containing peptides. The first search was conducted for maximum identification using the settings described above, and additional settings for detecting the heavy lysine Lys8, while the light label was set at unlabeled. Peptides that lack a lysine residue were selected and concatenated into “artificial proteins,” while respecting the position of the C-terminal amino acid. This newly created database with artificial proteins, the original databases containing *L. cremoris* proteins, and the frequently observed contaminants database were used in the second MaxQuant search for protein quantification with the protein quantification set to use unique peptides only. The peptides lacking a lysine residue were not identified as “unique” because they were represented in both the original *L. cremoris* database and the new artificial database. Consequently, only data obtained for peptides containing one or more lysine residues were used for protein quantification, allowing the accurate calculation of heavy and normal lysine-containing proteome fractions. For all MaxQuant searches, the nLC-MSMS system quality was checked with PTXQC (28) using the MaxQuant result files.

### Statistical analysis

The identified proteins were loaded into Perseus (version 1.6.15.0) and accepted when they were not only identified by site, did not match the reverse database, and had more than one peptide assigned, of which at least one must be unique to that protein. For long-term adapted proteomes, proteins were also removed when none of the conditions had the protein measured in at least two replicates. The LFQ values were log10 transformed, and missing values were imputed with randomly taken samples from a normal distribution with standard transformation settings. Proteomes were compared using fold changes per protein, and proteins with a fold change larger than fivefold were regarded as differentially expressed. Protein classes that were differentially expressed were identified per strain by gene set enrichment analysis using the clusterprofiler package (version 3.18.1) in R (version 4.0.2). Proteins were classified based on the higher categories of the Brite hierarchy, and protein classes were considered enriched at a *P*-value smaller than 0.05. To correlate the enriched classes with growth, the relative protein abundance of the total proteome was calculated by dividing each iBAQ value by the summed iBAQ values within a sample. The relative iBAQ abundances of proteins in enriched classes were summed and correlated with the measured growth rates.

For short-term adapted proteomes, the ratio between heavy and light proteins, as calculated by MaxQuant, was log transformed, and the slope and *y*-intercept were estimated. These protein turnover kinetic models were used to calculate the estimated time to reach equal levels of heavy and light proteins (log HL-ratio is 0; i.e., protein replacement time). As the proteins in the t0 sample did not contain heavy lysine yet, this sample was not included. The disappearance rate and production rate were calculated from the individual light and heavy proteins, respectively. To this end, relative iBAQ abundances were calculated to correct for sample variation and subsequently log transformed before estimating the slope over time for each protein for the light and heavy fractions separately (R; version 4.0.2).

To remove poorly modeled protein turnover relations, a model had to explain at least 70% of the variation and be based on at least four experimental observations. For models that did not meet these criteria, single outlier observations were removed when the observation-specific residual deviated more than the median residual of all observations in that model plus two times the residual median absolute deviation (mad) (29). Following outlier removal, newly fitted models were generated, which were only considered if the minimal model quality was reached (see above). Furthermore, replacement models of proteins that did not have at least four light and four heavy protein observations were discarded. Additionally, if a protein had less than four MaxQuant-calculated HL-ratios but passed the model criteria for both the disappearance (light-protein based) and the production (heavy-protein based) rate, the rate with the lowest median abundance was discarded. The distribution of the kinetic values (replacement, disappearance, and production) associated with accurately modeled proteins was examined, and protein half-lives that were bigger or smaller than the median half-life plus two times the half-life mad of all proteins were assigned as being faster or slower than growth.

## RESULTS

### Physiological and metabolic comparison of glucose- and galactose-adapted cultures

**Fig 1:**
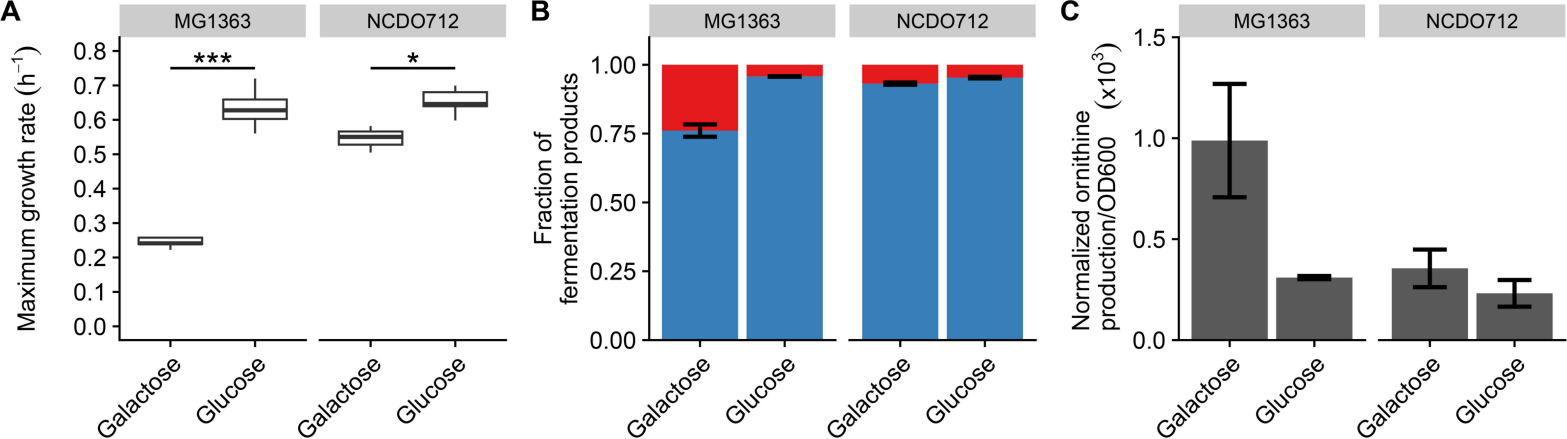
Physiological and metabolic profiles of long-term adapted *L. cremoris* NCDO712 and MG1363 grown on either glucose or galactose as the main carbon source. (A) Growth rates. (B) Fraction of total organic acid products for lactic (blue) and acetic (red) acid. (C) Normalized ornithine production per cell density (OD600). Error bars indicate standard deviation, while significance was determined with a two-sided Welch test and asterisks indicate ***P < 0.001, **P < 0.01, and *P < 0.05.

The main aim of this study was to investigate how *Lactococcus cremoris* accommodates proteome adaptation following an environmental shift, with a special focus on redundant proteins. We selected the environmental shift from galactose to glucose as the main carbon source, which has been associated with a large growth rate and physiological changes in *L. cremoris* MG1363 (30, 31). We also included its parental strain NCDO712 because its plasmids encode the *lac* operon that affects the growth on galactose (20, 32). Initial analyses focused on the physiological, metabolic, and proteome characterization of long-term adapted glucose- and galactose-grown *L. cremoris* cultures to define the difference under these growth conditions. To this end, both strains were grown in glucose- and galactose-supplemented media (25 mM) for at least 20 generations. The strains displayed a similar growth rate on glucose (~0.64 h^−1^; Fig. 1), which is comparable to previous reports (22, 33). Growth rate reduction on galactose was substantially smaller for NCDO712 (0.55 h^−1^) compared to MG1363 (0.24 h^−1^), again agreeing with previous reports (30, 31, 34–37) and confirming the role of the plasmid-encoded *lac* operon in galactose utilization in NCDO712 (32).

Metabolic differences between these cultures were analyzed by the relative abundances of various small molecules in the culture supernatant using NMR (Fig. 1B). Glucose-grown MG1363 displayed homolactic fermentation (>95%), whereas galactose-grown MG1363 displayed typical mixed acid fermentation (~75% lactic and 25% acetic acid), confirming earlier results (30, 31, 34–36). *L. cremoris* NCDO712 was also homolactic on glucose (>95%), but its higher growth rate on galactose relative to MG1363 was reflected by a marginal switch to mixed acid fermentation (~93% lactic and ~7% acetic acid). Notably, the acetic acid and lactic acid ratios are used here as a proxy for the relative distribution of pyruvate over the lactic- and mixed-acid pathways, because the concentrations of the additional metabolites that are formed in the latter pathway (e.g., formic acid and ethanol) could not reliably be determined due to their volatile nature. The *L. cremoris* strains used here encode the arginine deiminase (ADI) pathway that generates additional ATP by converting arginine to, among others, ornithine (34, 37) that is exchanged for extracellular arginine, indicating that extracellular ornithine serves as a proxy for ADI pathway activity. An approximately threefold higher ADI activity was observed in galactose- as compared to glucose-grown MG1363 (Fig. 1C), which was not observed in *L. cremoris* NCDO712, where ADI activity was unaffected by the carbon source and approximately equaled the level of glucose-grown MG1363.

Taken together, our results confirm that *L. cremoris* MG1363 has a lower growth rate, more mixed acid fermentation, and increased ADI activity when adapted to growth on galactose compared to glucose. These differences are also seen, but less pronounced, in its parental strain *L. cremoris* NCDO712.

### Matching physiology with proteome composition differences

**Fig 2:**
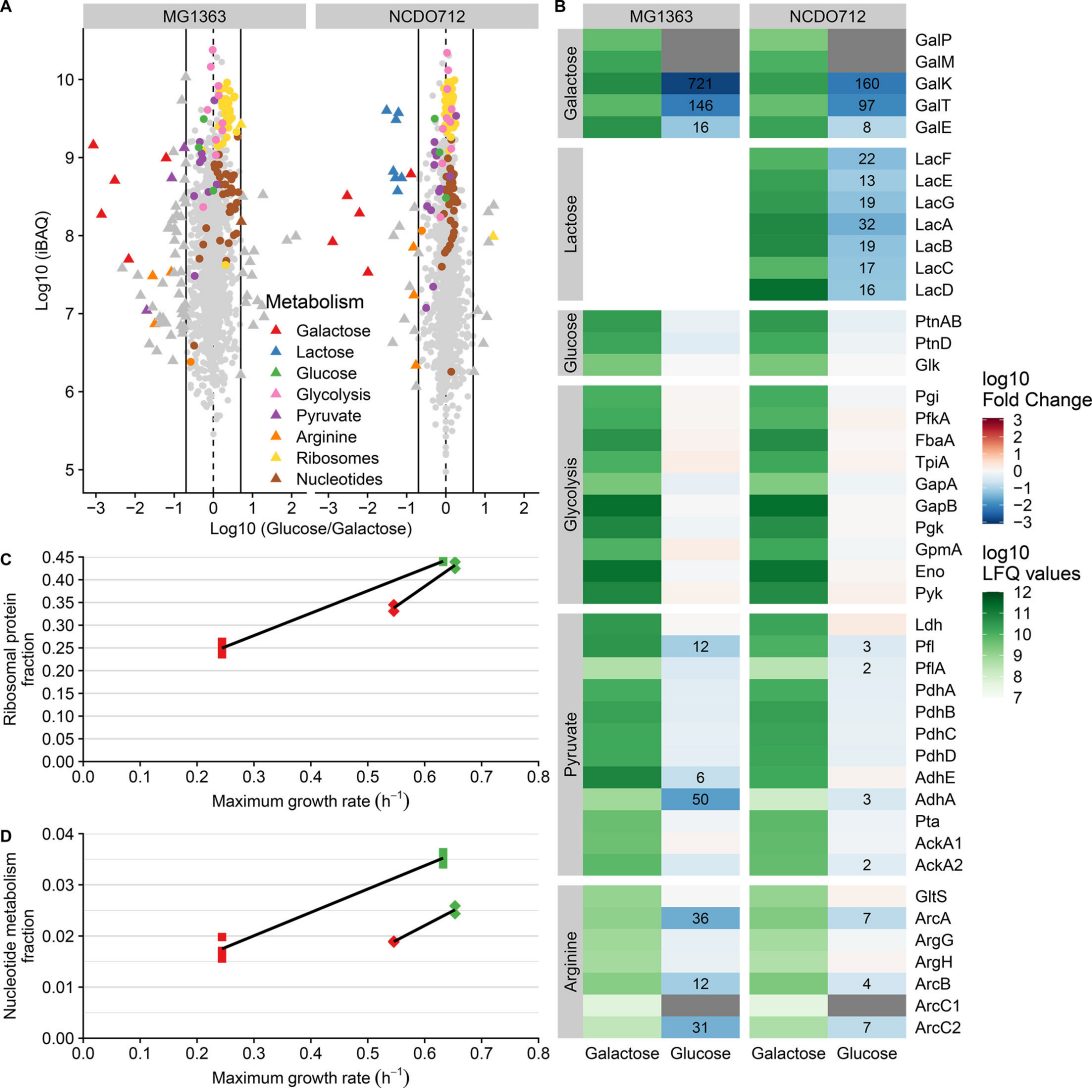
Differential proteome analysis in glucose- and galactose-adapted *L. cremoris* MG1363 and NCDO712. (A) Proteome comparison for both strains, where *x*- and *y*-axes indicate fold change between the two conditions and average expression level, respectively. Differentially expressed proteins (triangles) were identified using a fivefold difference cutoff (black lines). Proteins belonging to the most relevant pathways are colored. (B) Heatmap representation of the differentially expressed proteins assigned to pathways of interest, where shades of green indicate expression levels on galactose and the blue-red gradient indicates the fold change on glucose (cutoffs employed are fivefold for MG1363 and twofold for NCDO712; numbers in the blue cells indicate linear fold change), and gray indicates lack of detection in glucose cultures. (C and D) Proteome fraction (relative abundance using iBAQ values; *y*-axis) assigned to ribosomes (C) and nucleotide metabolism (D) correlated with growth rates (*x*-axis) for *L. cremoris* MG1363 (squares) and NCDO712 (diamonds) adapted to glucose (green) or galactose (red).

The proteomes of galactose- and glucose-grown cultures were compared to evaluate to what extent they reflect the observed differences in physiology. Proteome analysis identified and quantified more than 1,300 proteins per sample, corresponding to more than 50% of the genome-based proteome prediction. Differential protein expression analysis revealed 74 and 32 differentially expressed proteins between galactose- and glucose-grown cultures of *L. cremoris* MG1363 and *L. cremoris* NCDO712, respectively (Fig. 2A).

The most prominent difference between galactose- and glucose-grown proteomes was the expression of Leloir pathway proteins (Gal) that are involved in galactose import and metabolism (Fig. 2B). Both strains highly expressed Gal proteins on galactose, whereas these were 100- to 1,000-fold lower expressed (GalKT) or undetected (GalPM) on glucose. The exception was the UDP-galactose epimerase (GalE) that was only modestly downregulated (8- to 16-fold) on glucose, which is in agreement with the independent transcription of *galE* and its general housekeeping function (38, 39). The fold change of Gal protein levels was consistently larger in MG1363 compared to NCDO712, which likely reflects the contribution of the plasmid-borne *lac* operon to galactose import and metabolism in NCDO712 (20, 40). The *lac* operon (41) proteins were expressed in NCDO712 both when grown on galactose and glucose, albeit approximately 20-fold lower on glucose (Fig. 2B).

In contrast to the prominent proteome adaptation associated with growth on galactose, glucose growth adaptations were less obvious. For example, the main glucose import system, mannose PTS (PtnABD) (42), was equally expressed under the compared conditions (Fig. 2B). Likewise, no difference in the expression of the glycolytic pathway enzymes or lactate dehydrogenase (Ldh) was detected, while growth on glucose requires a substantially higher glycolytic flux to support faster growth on this sugar compared to galactose (35). Glucose-grown MG1363 expressed lower levels of pyruvate formate lyase (Pfl) and alcohol dehydrogenase (AdhA and AdhE), and especially, the reduced expression of the pivotal enzyme in the mixed acid fermentation pathway (Pfl) agrees with the reduced mixed acid production during glucose growth in this strain. Notably, the less pronounced shift toward mixed acid fermentation during galactose growth in NCDO712 is reflected by the much smaller fold changes in these proteins in this strain. Analogously, the higher activity of the ADI pathway in galactose-grown *L. cremoris* MG1363 was reflected by a 10- to 30-fold increased expression of the arginine catabolism enzymes (ArcABC1C2) (Fig. 2B). Again, in galactose-grown *L. cremoris* NCDO712, these enzymes were only modestly increased (four- to sevenfold), agreeing with the marginal differential ADI activity in this strain.

We also investigated the differential expression of proteins related to growth rate. In both *E. coli* (5) and *L. cremoris* (34, 43), higher growth rates have been demonstrated to be positively correlated with ribosome abundance. Gene set enrichment analysis (Fig. S1) revealed that the increased growth rate of MG1363 on glucose correlated with the increased expression of the functional classes “translation” (ribosomal proteins) and “nucleotide metabolism” (Fig. 2C and D), although individual proteins of these functional classes were not significantly differentially expressed. Conversely, “carbon metabolism”-associated proteins were higher expressed in galactose-grown cultures, corresponding with the high expression of the Gal proteins as well as the elevated expression of several other carbohydrate utilization functions due to relieved carbon catabolite expression under these conditions. Similar results were also obtained in *L. cremoris* NCDO712, albeit at a more modest level that reflects the substantially smaller growth rate effect seen in this strain (Fig. 2C and D).

Collectively, these results indicate that metabolic and growth rate differences observed in galactose- and glucose-grown cultures are reflected by proteome adaptations, corroborating previously reported proteome adaptations associated with carbon source-mediated growth rate changes in *L. cremoris* NCDO712 (43) and highlighting that these adaptations are amplified in its plasmid-free derivative MG1363.

### Proteome adjustment following a shift from galactose to glucose growth

The differences in proteome composition between glucose- and galactose-adapted cultures illustrated that the abundance of various proteins must be adjusted to completely adapt after a shift to a new sugar. However, these steady-state proteomes do not necessarily provide information on the dynamic process of such proteome adjustment. Therefore, we investigated short-term proteome adaptation in *L. cremoris* MG1363 and NCDO712 after an acute shift from galactose to glucose growth using proteome turnover analysis by stable isotope labeling. To this end, cultures growing in a medium with galactose and ^12^C^14^N lysine (“light”) were shifted to a medium with either glucose (“carbon shift”) or galactose (“control shift”) and ^13^C^15^N lysine (“heavy,” resulting in an additional 8 Da per lysine), and the proteome composition was determined over time. The ratio change between the heavy- and light-labeled proteins over time allowed for the estimated protein-specific replacement time (time point where heavy protein abundance equals light protein abundance) for more than 650 proteins after the medium shift.

#### Instant growth acceleration and proteome-wide adaptation following upshift to glucose

**Fig 3:**
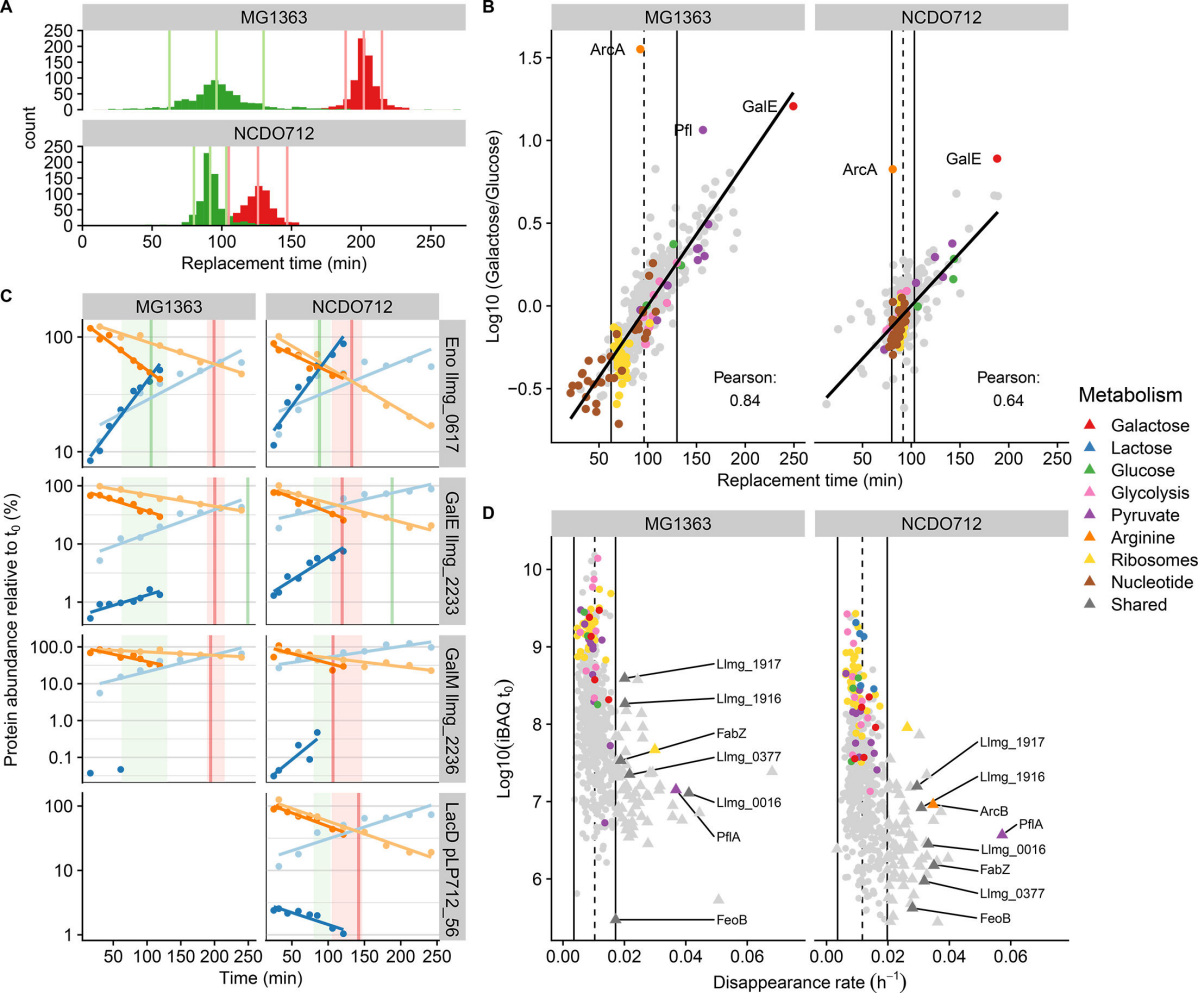
Proteome adaptation after the shift from galactose to glucose for *L. cremoris* NCDO712 and MG1363. (A) Distribution of replacement time (defined as time to replace the pre-shift protein abundance with the post-shift protein abundance) for both strains shifted to either glucose (green) or galactose (red). The middle vertical lines indicate the median replacement time as a proxy for the doubling time, and the two outer lines indicate the error margin for this doubling time. (B) Correspondence between replacement time (*x*-axis) and differential protein levels in adapted cultures (*y*-axis), where the vertical lines indicate the proxy for the doubling time. Proteins are colored based on their membership to relevant pathways. (C) Individual protein production (blue) and protein disappearance (orange) kinetics for four exemplary proteins, represented by the abundance of heavy-lysine and light-lysine protein abundances relative to time point zero (*y*-axis; log scale) over time (*x*-axis). Darker and lighter colored lines represent production and disappearance kinetics after the shift to glucose and galactose (control), respectively. Vertical lines represent the protein-specific replacement time following shift to glucose (green) and galactose (red), whereas the similarly colored vertical quadrants represent the doubling time approximation for these cultures. Notably, the initial time points following the slow-growing control shift (galactose to galactose) display an underestimation of the heavy protein abundance for many proteins, which is due to their close to the detection limit levels. (D) Disappearance rate per hour for proteins originating from the galactose environment (*x*-axis) plotted against the average protein expression prior to the carbohydrate shift (*y*-axis). Proteins are colored based on their membership to relevant pathways. Gray triangles represent proteins that are potentially actively degraded (i.e., disappearance rates higher than dilution by growth), and the named triangles represent proteins actively degraded in both strains.

Based on previous research on environmental shifts in *E. coli* (16), we assumed that most proteins are replaced as a function of growth. Therefore, the median replacement time was used as a proxy for the generation time. Strikingly, the estimated generation time after the shift to glucose was quite similar for both strains (96 and 92 minutes for MG1363 and NCDO712, respectively; Fig. 3A), whereas generation times after the control shift to galactose were similar to those observed during steady-state growth on this sugar (202 and 126 minutes for MG1363 and NCDO712, respectively; Fig. 3A).

The shorter generation time after the shift to glucose indicated an instantaneous acceleration of growth following the upshift in sugar quality, which is in agreement with similar protein levels observed for glucose import, glycolysis, and lactate dehydrogenase in both glucose- and galactose-adapted proteomes in both strains (Fig. 2B). Moreover, growth acceleration was supported by increasing ribosomal protein fractions that reached levels observed for glucose-adapted cultures within one generation time (Fig. S2).

To obtain a refined view of proteome adaptation following the shift to glucose, we correlated protein-specific replacement times with their differential abundance values observed in galactose- and glucose-adapted cultures (Fig. 3B). This analysis revealed strikingly high correlations for both strains, demonstrating that cells instantly initiate a proteome-wide adaptation following the shift from galactose to glucose. Notably, ribosomal and nucleotide metabolism-associated proteins consistently had replacement times that were shorter than the estimated generation time, which agrees with their higher abundance in the glucose-adapted cultures. Additionally, reduced expression in glucose-adapted cells corresponded well with replacement times that were longer than the estimated generation time for proteins involved in pyruvate dissipation, carbon metabolism, and ribosomal hibernation.

Only a few proteins appeared to deviate substantially from this correlation. A striking example in both strains is the ArcA protein (catalyzes the first step of the ADI pathway) that is expressed at a lower level in glucose- compared to galactose-adapted cultures, particularly in MG1363 (Fig. 2B). However, the ArcA protein replacement time was approximately equal to the estimated generation time, suggesting that the expression of this protein is maintained at the level observed in galactose-adapted cells during the first generation after the shift to glucose. In *L. cremoris* MG1363, the pyruvate formate lyase (Pfl) replacement time was substantially shorter than expected based on its differential expression in glucose-adapted cells. However, Pfl replacement time in *L. cremoris* NCDO712 was excluded from analysis because its replacement time was shorter in both the glucose shift, as well as the control galactose shift (Fig. S3), which suggests that replacement times for Pfl are confounded by its sensitivity to oxygen exposure (44) during culture-handling steps involved in the medium shift.

Overall, this analysis demonstrates that after a shift from galactose to glucose (carbon upshift), *L. cremoris* instantaneously accelerates its growth rate and immediately initiates proteome-wide adaptation.

#### Leloir and tagatose proteins (mainly) disappear by growth and are not replaced

The differential proteome analysis of glucose- and galactose-adapted cultures highlighted the Leloir (Gal), Tagatose (Lac), and ADI (Arc) pathways as the most differentially expressed (Fig. 2). Except for GalE and ArcA, the proteins associated with these pathways were not assigned a replacement time after the shift to glucose (Fig. 3B), while they were replaced at a rate corresponding with the estimated generation time after the control shift to galactose (Fig. S3).

To further explore the fate of these redundant proteins, we analyzed their protein production (heavy-labeled) and protein disappearance (light-labeled) rates separately. This analysis revealed that, in contrast to proteins that were replaced at a rate that follows growth (e.g., the glycolytic enzyme Eno; Fig. 3C), no replacement time could be calculated because protein production stagnated almost immediately after the shift to glucose (e.g., GalM; Fig. 3C) or was continued for only a short period followed by a stagnation of production (e.g., LacD; Fig. 3C).

To further unravel how these redundant proteins disappear from the proteome, we calculated the disappearance rate of all proteins based on the slope of the decreasing abundance of the light-labeled proteins over time (Fig. 3D). Reliable disappearance rates could be calculated for 649 proteins from *L. cremoris* MG1363 and 588 proteins from *L. cremoris* NCDO712. Analogously to the replacement time, we defined disappearance due to dilution by growth within two times the median absolute deviation relative to the median disappearance rate. These analyses established that the Gal and Lac proteins consistently dilute by growth (Fig. 3D). Unfortunately, except for ArcB in *L. cremoris* NCDO712 that disappeared faster than growth, we could not reliably calculate the disappearance rate of other Arc proteins because their disappearance rate modeling did not meet our model-quality standards. In addition, the heavy lysine-labeled Lac proteins that were initially produced after the shift to glucose appeared to subsequently decrease in abundance (e.g., LacD in Fig. 3C) at a rate that corresponded to dilution by growth. These analyses show that the production of most proteins that become redundant after the glucose shift stagnates instantly or shortly after the shift and that these pre-shift proteins disappear from the proteome through dilution by growth.

#### Several proteins involved in pyruvate dissipation are actively degraded

Although most proteins dilute by growth, some proteins displayed a significantly higher disappearance rate (Fig. 3D). In *L. cremoris* MG1363 and NCDO712, respectively, 59 and 66 proteins disappeared faster than dilution by growth after a shift to glucose but not after the control shift to galactose (Fig. 3D). These proteins belonged to different functional categories (Table S1) and in many cases were present prior to the shift at relatively low abundance, which poses challenges for accurate protein quantification and compromises disappearance rate modeling. As a consequence, a relatively large proportion of these proteins disappeared faster than dilution by growth in only one of the two strains (21%–30%) or could not be assessed reliably in both strains (50%–65%). Nevertheless, seven proteins had a consistently increased disappearance rate in both strains. These proteins include the putative GTP-driven iron uptake system (FeoB), the unsaturated fatty acid biosynthesis protein (FabZ), and two proteins with poorly defined functions that are possibly involved in cell division (llmg_0016) and competence regulation (llmg_0377). Strikingly, the remaining three proteins (PflA, llmg_1916, and llmg_1917) are all associated with pyruvate dissipation pathways. PflA is the activator for Pfl that is responsible for pyruvate to acetyl-CoA and formate conversion and is a key determinant in mixed acid carbon flux. The proteins encoded by *llmg_1916* and *llmg_1917* are predicted to form a complex with the *llmg_1915*-encoded protein (diluted by growth) that has been postulated to convert lactate into pyruvate (45, 46). Importantly, these proteins were found to be nine- and twofold lower expressed in glucose- compared to galactose-adapted cells of *L. cremoris* MG1363 and *L. cremoris* NCDO712, respectively, which agrees with the observed adjustment of their production and disappearance rate during the carbon shift.

Remarkably, both the *llmg_1916*-encoded protein and PflA depend on a 4Fe-4S cluster as a cofactor, which inspired us to evaluate the disappearance rate of the 15 additional proteins that have been proposed to depend on the 4Fe-4S cluster in *L. cremoris* MG1363 and NCDO712. Nine of these proteins were detected in our proteome analyses, and for six of those proteins, the disappearance rates could reliably be calculated (fulfilling our model quality requirements) in MG1363. Strikingly, five of these proteins disappeared at a rate higher than dilution by growth, while the sixth protein (NifJ) tended to disappear at an accelerated rate but not significantly higher than dilution by growth. Unfortunately, in NCDO712, the disappearance rates of only three of the proposed 4Fe-4S cofactor-dependent proteins could be reliably assessed, which included besides the above-mentioned PflA and Llmg_1916, also the NifJ protein. These findings support that 4Fe-4S-containing proteins are actively degraded following the environmental shift from galactose media to glucose media. Active removal of PflA and Llmg_1916 (part of the postulated lactate utilization complex) is likely to accelerate adaptation toward the glucose-growth-associated homolactic fermentation, rather than the mixed-acid fermentation that is characteristic for growth on galactose.

## DISCUSSION

After an environmental shift, bacteria adapt their proteome composition to optimize their growth and fitness. Here, we investigated how *Lactococcus cremoris* accommodates proteome-level changes during an environmental shift by investigating protein turnover after shifting the culture from galactose to glucose as the main carbon source. Contrary to most studies regarding proteome changes following an environmental shift, the carbon shift we studied is considered a nutrient upshift that lacks a transition or lag phase and is not confounded by the residual presence of the pre-shift carbon source (8, 10, 16), allowing us to investigate the fate of proteins that become redundant after the shift in detail. Notably, growth on galactose or glucose in *L. cremoris* presents a well-established example of contrasting carbohydrate substrates that impact growth rate, glycolytic flux, and fermentation characteristics (homolactic versus mixed acid) (30, 31). We confirmed these physiological differences and highlighted that they are more pronounced in the plasmid-free strain MG1363 compared to its parental strain NCDO712, which contains various plasmids. These physiological differences are largely reflected by the proteome composition, including the reduced expression of the ADI and mixed-acid pathways in glucose-grown cells, and the increased expression of ribosomal proteins and nucleotide metabolism proteins, which were also more pronounced in MG1363 than in NCDO712. These proteome data confirm previous results that determined individual protein levels in cultures grown under similar conditions (31) and agree with proteome adaptations in cells growing at different rates in glucose-limited chemostats (34, 47).

We also demonstrate that a galactose-grown culture immediately accelerates its growth and protein replacement time in response to the shift to glucose, which is in agreement with the immediate growth rate increase observed when adding glucose to a galactose-growing culture (48). Our proteome analyses support that this acceleration is facilitated by the glucose import and glycolysis-associated proteins that are equally abundant in galactose- compared to glucose-grown cultures. This finding agrees with our previous finding that proteome compositions of *L. cremoris* NCDO712 grown on various carbon sources predict the “preparedness” of cells to catabolize alternative carbon sources (43), which resembles similar conclusions reached in *E. coli* (16).

The growth rate adjustment is accompanied by an immediately initiated and proteome-wide adjustment toward a glucose-adapted proteome. It should be noted that this adjustment is not immediately achieved after the shift, indicating that the growth rate is probably accelerating during the first generation(s) following the glucose shift. During this accelerating growth, the translation capacity rapidly increases due to the enhanced synthesis rate of ribosomal proteins, suggesting that an almost complete growth rate adjustment is accomplished within the first generation after the substrate upshift. This suggestion is in agreement with the very similar generation time estimated for the two strains following their shift to glucose, even though their original growth rate on galactose differed substantially.

Our data demonstrated that proteome adaptation is mainly guided by protein production rather than degradation since most proteins disappear from the cell through dilution by growth. The instant proteome-wide adaptation in response to glucose is likely resulting from instant and large-scale transcriptome adaptation that is orchestrated by global regulators. Such a global regulation strategy during adaptation was previously proposed for *E. coli*, involving global regulators of carbon catabolite repression (cAMP) and ribosome synthesis (ppGpp), potentially combined with competition for transcriptional and translational resources (16). Although carbon catabolite repression and ppGpp-mediated regulation differ between *E. coli* and Firmicutes like *L. cremoris* (49, 50), the reduced replacement time of proteins involved in carbon metabolism and accelerated replacement time of ribosomal proteins suggest the involvement of similar mechanisms in the instantaneous adaptation in *L. cremoris*. However, in contrast to the group-based proteome adjustment analysis in *E. coli*, our correlation approach considers all proteins, resulting in an unprecedented resolution, and reveals a proteome-wide adaptation that implies the involvement of additional regulatory systems and exemplifies that the regulatory mechanisms that control proteome-wide adaptation remain to be further elucidated.

The production-guided proteome adaptation also applies to the adaptation of (partially) redundant protein levels, where reduced production and dilution by the growth of excess protein levels drive the protein-level adjustment. Notably, we could not assess the precise fate of various arginine metabolism proteins since they were also affected in the control shift (galactose to galactose), suggesting that they were affected by the culture-handling procedures during the shift. The arginine metabolism is subject to complex regulation in *L. cremoris*, involving many regulators (ArgR, AhrC, the sRNA ArgX, CcpA, and CodY) that respond to a variety of environmental cues and target different *cis*-acting elements in the *arc* operon that is transcribed in several different transcripts (37, 51–56). Under the conditions we employed, our results remain inconclusive concerning the mechanisms underlying ADI pathway adjustment, but similar experimental approaches using ArgR and AhrC deficient (52) or ArgX overexpressing (56) derivatives of strain MG1363 may further decipher the mechanisms involved.

Besides partially redundant proteins, proteins that become completely redundant after the shift to glucose are also adjusted through production-guided adaptation. Consequently, these proteins are not actively removed, and the residual protein levels will likely shorten the lag phase if cells are returning to a galactose-containing environment within the first generation(s) after the shift to glucose. This reduction of the lag phase has been termed “metabolic memory” and has previously been described for *E. coli* growing in a dynamic environment that alternates between glucose and lactose as substrates for growth (10).

The proteins of the plasmid-encoded tagatose pathway in the NCDO712 strain are an apparent exception to the production-guided adjustment strategy. These Lac proteins are required for lactose utilization but also contribute to galactose metabolism (32) and were expressed at a lower level in the glucose-adapted cultures. However, rather than adjusted production levels after the shift from galactose to glucose, the production of these proteins completely stagnated shortly after the shift, and the Lac proteins present in the cell disappeared from the proteome through dilution by growth. This aberrant finding may be explained by the loss of the co-inducer tagatose-6-phosphate (57) that would lead to LacR-mediated repression of *lac* operon transcription (58). Due to autoregulation of LacR (58), subsequent growth on glucose would lead to dilution by growth of the LacR protein, which below a certain threshold could relieve *lac* operon repression and regain of Lac protein production to the level observed in glucose-adapted cells.

Although most (partially) redundant proteins dilute by growth, we identified seven proteins that were actively degraded after the shift to glucose in both strains. Notably, the peptide profiles underlying the quantifications of these actively degraded proteins did not reveal site-specific cleavage or other molecular indicators (e.g., amino acid modifications) that facilitated their recognition by cellular protein degradation machineries (e.g., the Clp protease system). One of these proteins is the pyruvate formate lyase activator (PflA) that activates the gateway enzyme toward mixed acid fermentation Pfl by introducing a free radical on a glycine residue near the C-terminus of Pfl (59). Following the shift to glucose, PflA production immediately stagnates, and the protein present in the cell is actively degraded, which indicates that despite the continued production of Pfl, its activity is lost due to rapidly declining activation by PflA. Notably, the Pfl protein present in the galactose-grown preculture is likely inactivated due to oxygen exposure during culture handling steps involved in the galactose to glucose shift (44). Consequently, pyruvate dissipation toward acetyl-CoA and mixed acid fermentation is rapidly shut down and reoriented to homolactic fermentation that is associated with glucose growth. Strikingly, besides PflA, two proteins of a protein complex that is proposed to convert lactate into pyruvate (45, 46) were also actively degraded. This conversion of lactate to pyruvate may contribute to ATP generation during growth on galactose by increasing the pyruvate availability for acetate production that generates an extra ATP but would counteract lactate production in glucose environments. Taken together, these rare observations of active protein degradation after the shift to glucose are likely contributing to an accelerated reorientation of pyruvate dissipation toward homolactic fermentation.

Inspired by the notion that both PflA and the lactate utilization complex rely on a 4Fe-4S cluster as a cofactor, we established that (virtually) all detected and quantified 4Fe-4S-dependent proteins predicted in *L. cremoris* were actively degraded after the shift to glucose. This indicates that the loss of this co-factor possibly results in the unfolding of these proteins, inducing their active degradation. Although the 4Fe-4S co-factor is highly oxygen sensitive (60), the postulated loss of this co-factor is not explained by oxygen exposure during the culture handling steps involved in the shift, because the control to galactose did not coincide with active degradation of these 4Fe-4S-dependent proteins. This co-factor loss could result from disturbed iron homeostasis, which is supported by the active degradation of FeoB (Fe^2+^ importer) after the shift to glucose. These findings suggest that 4Fe-4S co-factor binding and iron homeostasis play a generic role in stabilizing proteins that bind this co-factor, including several proteins that regulate the metabolic switch between mixed-acid and homolactic pyruvate dissipation.

Although our results contribute to the understanding of the mechanism underlying the shut down of mixed acid fermentation following a shift to glucose, our mechanistic understanding of this metabolic switch in *L. cremoris* remains incomplete (61). For example, the continued expression of most of the mixed-acid pathway proteins during homolactic glucose growth has been a topic of interest for many years (61), and studies implicating redox balance and glycolytic flux (35, 36, 61) were unable to provide a full explanation for the control of the metabolic switch. Our work enriches this mechanistic view by adding a pivotal role for PflA in Pfl activity control, where we propose that PflA, in turn, is controlled by iron homeostasis and 4Fe-4S co-factor availability. Thereby, the control of pyruvate dissipation through mixed acid versus homolactic fermentation appears to involve various intertwined mechanisms, which underpins the importance of strictly controlling this metabolic switch in the lifestyle of *L. cremoris*.

## Data Availability

The mass spectrometry proteomics data have been deposited to the ProteomeXchange Consortium via the PRIDE partner repository with the data set identifiers PXD042113 (ftp download: https://ftp.pride.ebi.ac.uk/pride/data/archive/2025/02/PXD042113) and PXD041822 (ftp download: https://ftp.pride.ebi.ac.uk/pride/data/archive/2025/02/PXD041822).

## SUPPLEMENTAL MATERIAL

Supplemental material mbio.02990-24-s0001.pdf Figures S1 to S3; Table S1.

ASM does not own the copyrights to Supplemental Material that may be linked to, or accessed through, an article. The authors have granted ASM a non-exclusive, world-wide license to publish the Supplemental Material files. Please contact the corresponding author directly for reuse.
