# Supplementary material for: Carbon upshift in Lactococcus cremoris elicits immediate initiation of proteome-wide adaptation, coinciding with growth acceleration and pyruvate dissipation switching
Source: mBio. 2025 Feb 20;16(3):e02990-24. doi: 10.1128/mbio.02990-24 (PMC11898756; doi:10.1128/mbio.02990-24)
Supplement: Supplemental material — Figures S1 to S3; Table S1. [file mbio.02990-24-s0001.pdf]

Supplemental Material

For:

Carbon upshift in *Lactococcus cremoris* elicits immediate initiation of proteome-wide adaptation, coinciding with growth acceleration and pyruvate dissipation switching

Berdien van Olst, Sjef Boeren, Jacques Vervoort, Michiel Kleerebezem

Supplemental Figures

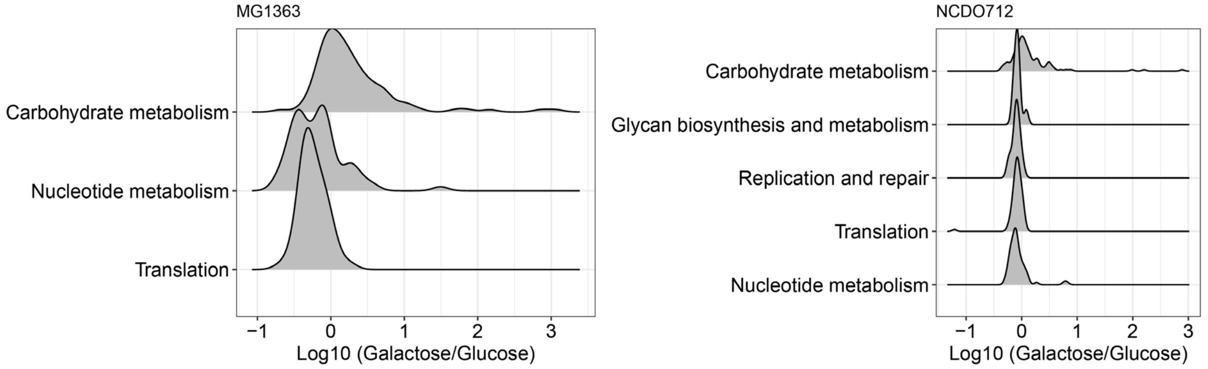

Supplemental Figure SF1: Distributions of Brite-defined protein classes (y-axis) that are enriched in either glucose (negative values) or galactose (positive values) adapted proteomes (x-axis) for both strains (panels).

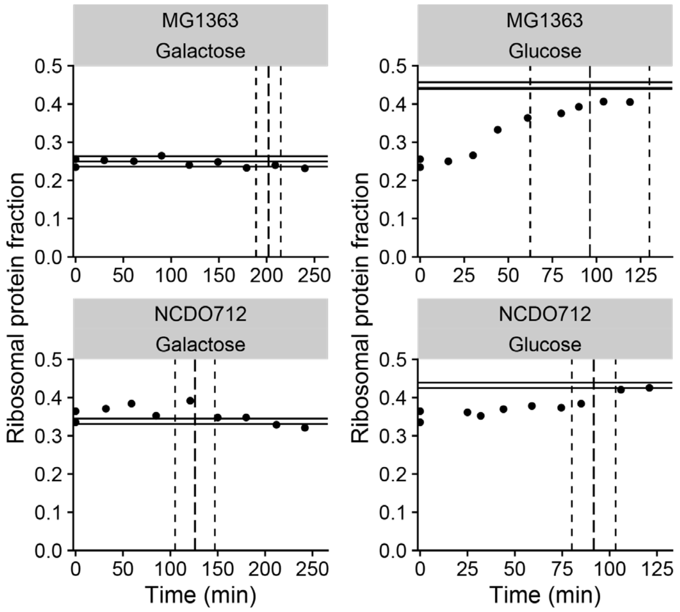

Supplemental Figure SF2: Ribosomal fraction of the total proteome (y-axis) over time (x-axis) after the carbon shift to glucose or the control shift to galactose (horizontal panels) for the two strains (vertical panels). The horizontal lines indicate the ribosomal fraction in steady-state conditions for two (NCDO712) or three (MG1363) replicates, whereas the vertical lines indicate the median replacement time as proxy for the doubling time (long dashes) and the error margin for this doubling time (short dashes).

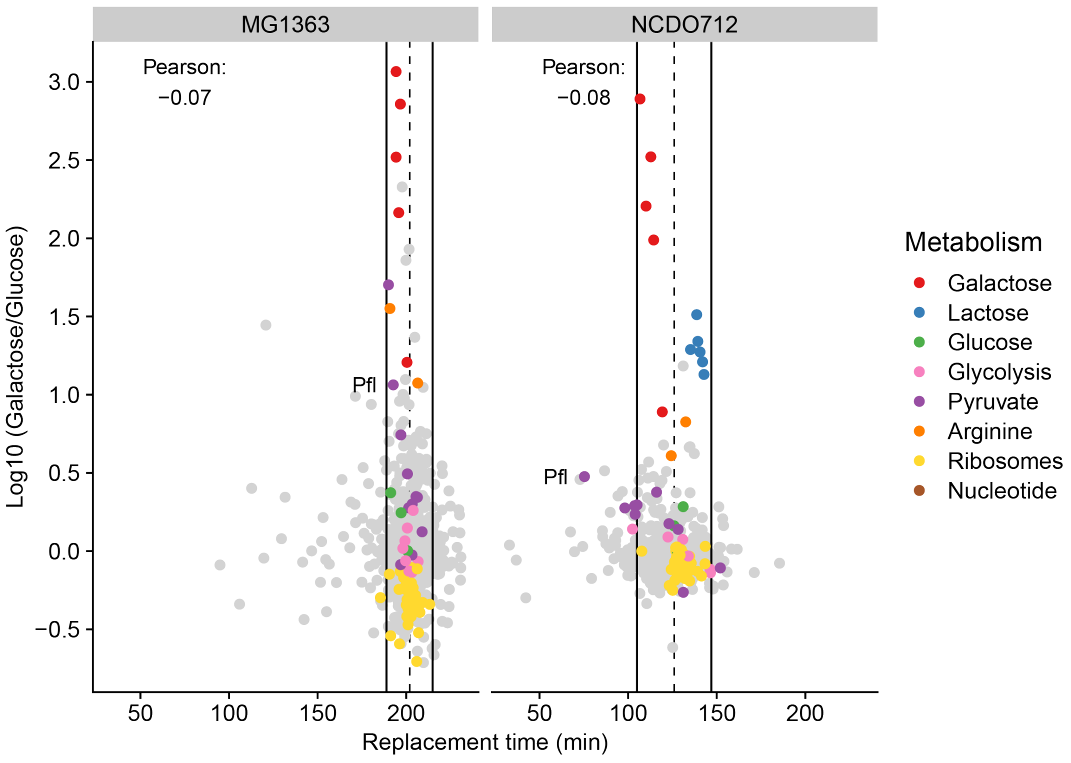

Supplemental Figure SF3: Correspondence between short-term and long-term proteome adaptation. The x-axis indicates the replacement time in minutes of the proteins after the shift to galactose (short-term adaptation), the y-axis indicates the fold-change expression between proteomes long-term adapted on galactose and glucose, the proteins are coloured based on their membership to important pathways and the vertical lines indicate the proxy for the doubling time.

Supplemental Table ST1: Proteins that disappear faster than growth after the shift to glucose but not after the control-shift to galactose in either of the strains.

	MG1363			NCDO712			
Uniprot ID	Estimated disappearance rate (h-1)	Classified disappearance rate	log10 iBAQ t0	Estimated disappearance rate (h-1)	Classified disappearance rate	log10 iBAQ t0	Gene name
A2RH88	0.041	Faster	7.110	0.033	Faster	6.450	llmg_0016
A2RHF4	0.011	Not faster	7.277	0.025	Faster	6.044	llmg_0084
A2RHG2	0.020	Faster	6.721	NA	NA	5.669	cysD llmg_0091
A2RHQ4	0.029	Faster	7.374	NA	NA	6.380	llmg_0185
A2RHR8	0.017	Faster	5.473	0.028	Faster	5.624	feoB llmg_0199
A2RHT0	0.025	Faster	7.205	NA	NA	6.102	rgpA llmg_0211
A2RHT8	0.008	Not faster	7.856	0.037	Faster	6.870	llmg_0219
A2RHU1	0.018	Faster	7.388	NA	NA	6.605	wefC llmg_0222
A2RHV6	0.039	Faster	6.759	NA	NA	5.771	llmg_0237
A2RHW2	0.010	Not faster	6.907	0.031	Faster	6.258	ung llmg_0244
A2RHX6	0.024	Faster	6.945	NA	NA	5.768	pepDA llmg_0260
A2RHY5	0.018	Faster	7.437	NA	NA	6.171	llmg_0270
A2RHZ5	0.026	Faster	7.962	NA	NA	6.941	acmA llmg_0280
A2RI39	0.010	Not faster	7.270	0.025	Faster	6.176	murA murA1 llmg_0326
A2RI88	0.022	Faster	7.351	0.032	Faster	5.972	llmg_0377
A2RIA9	0.051	Faster	5.727	NA	NA	NA	nha llmg_0399
A2RIC5	0.025	Faster	6.988	NA	NA	5.592	holB llmg_0416
A2RIC6	0.032	Faster	7.202	0.015	Not faster	5.846	llmg_0417
A2RIF6	0.014	Not faster	7.604	0.021	Faster	6.299	nifJ llmg_0447
A2RIH5	0.017	Faster	7.442	0.018	Not faster	5.993	aspC llmg_0466
A2RIN6	0.027	Faster	7.084	NA	NA	NA	llmg_0527
A2RIP2	0.019	Faster	6.912	NA	NA	5.772	nudH llmg_0533
A2RIP6	0.019	Faster	7.530	0.035	Faster	6.174	fabZ llmg_0538
A2RIS1	0.019	Faster	6.787	NA	NA	6.049	llmg_0564
A2RIU2	0.010	Not faster	8.640	0.020	Faster	7.222	llmg_0589
A2RIW0	0.020	Faster	7.955	NA	NA	7.551	apt llmg_0607
A2RIY1	0.007	Not faster	7.741	0.025	Faster	6.234	fadD llmg_0627
A2RIZ0	0.036	Faster	6.949	NA	NA	NA	citB llmg_0636
A2RJ77	0.020	Faster	7.180	0.012	Not faster	6.162	llmg_0724
A2RJ79	0.018	Faster	7.528	NA	NA	6.319	llmg_0726
A2RJ96	0.017	Faster	7.842	NA	NA	NA	agl llmg_0744
A2RJQ4	0.021	Faster	7.077	0.014	Not faster	6.146	murG llmg_0913
A2RJT0	0.010	Not faster	7.599	0.020	Faster	5.915	llmg_0941
A2RJU0	0.019	Faster	7.329	NA	NA	NA	llmg_0953
A2RK10	0.020	Faster	7.151	NA	NA	7.133	llmg_1025
A2RK13	-0.024	Not faster	7.026	0.028	Faster	6.537	llmg_1028
A2RK42	0.014	Not faster	6.695	0.022	Faster	6.071	rsmC llmg_1059

A2RKB7	0.021	Faster	6.634	NA	NA	5.448	sbcC llmg_1133
A2RKF4	0.028	Faster	NA	NA	NA	5.571	llmg_1171
A2RKG7	0.020	Faster	7.391	0.017	Not faster	6.601	gltD llmg_1184
A2RKI5	0.021	Faster	7.183	NA	NA	5.664	llmg_1203
A2RKK2	0.028	Faster	NA	NA	NA	NA	NA
A2RKM5	0.018	Faster	6.843	NA	NA	NA	arsR llmg_1246
A2RKV5	0.023	Faster	7.031	0.029	Faster	NA	niaX llmg_1330
A2RKW6	0.006	Not faster	7.835	0.024	Faster	6.375	dfrA llmg_1342
A2RKY1	0.018	Faster	6.926	NA	NA	6.038	llmg_1357
A2RL81	0.019	Faster	7.388	NA	NA	5.741	llmg_1468
A2RLA7	0.018	Faster	7.386	NA	NA	6.532	llmg_1496
A2RLB3	0.026	Faster	6.807	NA	NA	6.356	dnaD llmg_1502
A2RLF8	0.022	Faster	6.852	0.010	Not faster	5.922	llmg_1550
A2RLG3	0.068	Faster	7.387	NA	NA	7.167	whiA llmg_1555
A2RLK3	0.018	Faster	7.835	NA	NA	6.564	llmg_1597
A2RLX8	0.021	Faster	6.680	NA	NA	6.110	sdaA llmg_1732
A2RLY7	0.023	Faster	6.674	NA	NA	NA	llmg_1742
A2RM15	0.023	Faster	6.736	NA	NA	5.351	noxC llmg_1770
A2RM51	0.018	Faster	7.403	0.012	Not faster	6.346	llmg_1807
A2RMF5	0.020	Faster	8.266	0.031	Faster	6.921	llmg_1916
A2RMF6	0.020	Faster	8.593	0.029	Faster	7.199	llmg_1917
A2RMG9	0.021	Faster	6.694	NA	NA	6.146	llmg_1930
A2RMN3	0.037	Faster	7.153	0.057	Faster	6.569	pflA llmg_1997
A2RMP2	0.018	Faster	7.316	0.018	Not faster	6.637	uvrA llmg_2008
A2RMQ2	0.012	Not faster	7.781	0.024	Faster	7.091	llmg_2018
A2RMV5	0.011	Not faster	8.369	0.021	Faster	7.549	llmg_2074
A2RN42	0.021	Faster	7.209	0.019	Not faster	6.236	birA2 llmg_2162
A2RN64	0.010	Not faster	8.163	0.027	Faster	7.184	lacX llmg_2185
A2RN95	0.023	Faster	7.110	NA	NA	6.050	llmg_2216
A2RNA6	0.021	Faster	6.541	NA	NA	NA	llmg_2227
A2RNC0	0.009	Not faster	7.533	0.032	Faster	6.465	llmg_2243
A2RNJ2	0.027	Faster	6.738	NA	NA	4.910	snf llmg_2319
A2RNY5	0.028	Faster	6.455	NA	NA	5.604	recX llmg_2466
A2RNZ3	0.007	Not faster	7.820	0.020	Faster	7.162	ssbB llmg_2474
A2RP08	0.007	Not faster	7.236	0.025	Faster	5.912	mutL llmg_2489
A2RP33	0.036	Faster	6.803	NA	NA	5.866	llmg_2516
A2RP61	0.030	Faster	7.671	NA	NA	7.630	rpsI llmg_2545
A2RP78	0.015	Not faster	6.975	0.022	Faster	6.291	llmg_2562
P22865	0.026	Faster	8.138	NA	NA	7.570	usp45 llmg_2507
Q7BPI7	0.016	Not faster	7.037	0.022	Faster	6.010	kinD llmg_1649
Q9RLU5	0.031	Faster	7.148	NA	NA	NA	alr llmg_1704
A2RIP5	NA	NA	6.939	0.020	Faster	5.758	argE llmg_0536
A2RJB7	NA	NA	7.568	0.025	Faster	6.877	llmg_0765
A2RKA1	NA	NA	7.944	0.026	Faster	6.749	nagA llmg_1117
A2RN65	NA	NA	7.665	0.022	Faster	6.618	cbf llmg_2186
A2RNF9	NA	NA	7.662	0.020	Faster	6.726	llmg_2282

Q9K575	NA	NA	7.704	0.035	Faster	6.962	arcB llmg_2312
Q9L6G1	NA	NA	7.491	0.027	Faster	5.682	engB llmg_1339
A2RHE7	NA	NA	6.903	0.025	Faster	6.007	llmg_0077
A2RHH0	NA	NA	8.077	0.026	Faster	7.957	rpmF llmg_0099
A2RHT1	NA	NA	7.016	0.022	Faster	6.446	rgpB llmg_0212
A2RHV2	NA	NA	6.807	0.028	Faster	5.998	hflX llmg_0233
A2RI29	NA	NA	5.853	0.027	Faster	6.372	cpdC llmg_0316
A2RIB4	NA	NA	7.503	0.020	Faster	6.685	llmg_0405
A2RIH1	NA	NA	6.867	0.028	Faster	5.792	truA llmg_0462
A2RIQ1	NA	NA	7.553	0.023	Faster	6.431	dfpA llmg_0543
A2RIQ2	NA	NA	7.185	0.031	Faster	6.164	dfpB llmg_0544
A2RIV7	NA	NA	6.493	0.036	Faster	5.442	rnz llmg_0604
A2RIW8	NA	NA	7.476	0.040	Faster	6.267	ctsR llmg_0614
A2RIY7	NA	NA	6.894	0.027	Faster	6.465	ftsW1 llmg_0633
A2RJ86	NA	NA	6.747	0.020	Faster	6.116	llmg_0733
A2RJ98	NA	NA	6.020	0.020	Faster	6.527	malR llmg_0746
A2RL77	NA	NA	7.500	0.036	Faster	6.225	aldC llmg_1464
A2RLE0	NA	NA	7.373	0.023	Faster	7.152	ribB llmg_1531
A2RLX5	NA	NA	6.363	0.021	Faster	5.450	copA llmg_1729
A2RLY1	NA	NA	6.259	0.027	Faster	6.574	noxA llmg_1735
A2RM67	NA	NA	6.765	0.038	Faster	NA	ansB llmg_1823
A2RMN7	NA	NA	6.310	0.021	Faster	5.726	llmg_2003
A2RMP8	NA	NA	7.209	0.020	Faster	6.293	llmg_2014
A2RMS9	NA	NA	7.670	0.023	Faster	6.692	nifS llmg_2048
A2RMU3	NA	NA	7.522	0.035	Faster	6.755	llmg_2062
A2RNA4	NA	NA	6.857	0.027	Faster	6.402	llmg_2225
A2RNE2	NA	NA	NA	0.022	Faster	6.181	ps504 llmg_2265
A2RNE8	NA	NA	7.585	0.024	Faster	6.596	ecsA llmg_2271
A2RP29	NA	NA	7.291	0.026	Faster	6.709	rcfB llmg_2512
K7DWE7	NA	NA	NA	0.028	Faster	6.407	parA pLP712_22b
P42370	NA	NA	6.884	0.033	Faster	6.057	hrcA llmg_1576
P60376	NA	NA	6.741	0.030	Faster	6.198	spxA nrpR llmg_0640
pNZ712_19	NA	NA	6.540	0.033	Faster	6.293	lcoC pNZ712_19
Q7BGF0; A2RJ63	NA	NA	6.008	0.023	Faster	6.414	NA
